# Mechanical Behavior of GFRP Laminates Exposed to Thermal and Moist Environmental Conditions: Experimental and Model Assessment

**DOI:** 10.3390/polym14081523

**Published:** 2022-04-09

**Authors:** Getahun Tefera, Sarp Adali, Glen Bright

**Affiliations:** Discipline of Mechanical Engineering, University of KwaZulu-Natal, Durban 4041, South Africa; adali@ukzn.ac.za (S.A.); brightg@ukzn.ac.za (G.B.)

**Keywords:** glass/epoxy laminate, mechanical properties, temperature behavior, empirical models

## Abstract

This paper presents an experimental and analytical study about the mechanical response at a different temperature on glass fiber-reinforced polymer laminates. The effect of different environmental conditions on compressive, tensile, stiffness, and viscoelastic behavior (storage modulus, loss modulus and damping ratio) of laminates were investigated. Before testing, laminates were preserved in a deep freezer at −80 °C, −20 °C, 0 °C, and room temperature (25 °C) for up to 60 days. Results confirmed that temperatures ranging from −80 to 50 °C, which were below the glass transition temperature of the epoxy resin, did not significantly affect the compressive, tensile, and stiffness performance of all laminates. When the testing temperature increased to 100 °C, the properties were decreased significantly due to the damaging of the fiber/matrix interface. Additionally, results obtained from dynamic mechanical analyses tests showed a drop-in storage modulus, high peaks in loss modulus and high damping factor at the glass transition region of the epoxy resin. The highest storage modulus, two phases of glassy states and highest damping ratio on the −80/G group of laminates were obtained. The accuracy of experimental results was assessed with empirical models on the storage modulus behavior of laminates. The empirical model developed by Gibson et al. provided accurate estimates of the storage modulus as a function of temperature and frequency. The remaining empirical models were less accurate and non-conservative estimations of laminates stiffness.

## 1. Introduction

In recent years, fiber-reinforced polymer (FRP) composite materials have been widely used for structural applications, especially in the fields where lightweight, high strength and high durability were required [1,2,3]. Among fibrous materials, glass fiber has good insulation, high mechanical strength, low cost, strong heat and corrosion resistance behavior. These characteristics are ideal as a reinforcing material in marine and wind turbine blade structural components [4,5]. The development of FRP composite materials for large wind turbine blades, reinforcing bars, and cyclically loaded structures are often exposed to hot and cold environmental conditions, during their service life. The mechanical properties of composite structures need considerations under variable environmental working conditions [6]. Mainly, glass fiber-reinforced polymer (GFRP) are being a candidate material and is increasingly used for the structural design of wind turbine blades and reinforced bars in civil engineering applications. Stiffness, strength and bonding behavior of materials are severely affected at elevated temperatures, approaching the glass transition temperature of the polymer matrix [7,8]. It needs further tests to assess the properties of GFRP material under different environmental conditions.

Mathiev and Brahim [9] investigated the mechanical properties of GFRP bars subjected to extreme temperatures. Their experiments used sand-coated GFRP bars exposed to low temperatures from 0 to −100 °C and high temperatures from 23 to 315 °C. Flexural, shear and tensile strength tests were carried out. Results indicated that flexural, shear and tensile strength of GFRP bars increased when testing temperature decreased. This change in mechanical behavior occurred when a high level of moisture was contained in the material that initiated microcracks. For the case of higher temperature tests, flexural, shear and tensile strength of GFRP bars were decreased.

Regarding humid environmental conditions, Ellyin and Rohrbacher [10] performed an experimental investigation on the absorption behavior of GFRP laminates immersed in distilled water. They looked mechanical behavior of laminates at ambient and 90 °C temperatures. A tensile test was performed on three laminate lay-ups in three different environments. It was found that the strength and durability of GFRP laminates decreased due to immersion in 90 °C of water. The rate of moisture absorption of laminates was highly dependent on immersed water temperatures. Mainly, immersion at high water temperatures leads to brittle failure modes. Additionally, cross-ply, multidirectional and angle-ply laminates were immersed in distilled water for four months. A steady-state moisture uptake was reached at ambient temperature, while no such state of saturation was observed for GFRP laminates immersed at 90 °C. Fatigue life curve was observed in dry and immersed laminates. Results indicated that immersion in 90 °C caused high cracking and a reduction in fatigue resistance.

Faster moisture uptake on FRP materials develops faster mechanical degradation behavior. It is required to assess the moisture absorption behavior in a composite laminate. Lundgren and Gudmundson [11] investigated the moisture uptake behavior of cross-ply glass-fiber/epoxy laminates containing matrix cracks in transverse plies. Both experimental and finite element results indicated that moisture swelling causes cracking in the moisture absorption process of GFRP laminates. Additionally, important to assess the failure properties of GFRP materials under tensile and compressive loading before using them for designing columns in marine and wind turbine blade structures [12]. Regarding compression behavior, Wong et al. [13] investigated numerical and experimental study on compression properties of short glass-reinforced plastic C-shaped channels at elevated temperatures. Results showed that the compressive strength of C-channels material becomes highly dependent on softening behavior of the polymer matrix. In the earlier study, the temperature-dependent mechanical behavior of FRP material applicable to the manufacturing of wind turbine blade structures was investigated by different authors [14]. Additionally, temperature effects on mechanical properties of glass/thermoplastic laminates were evaluated [15]. The results of the author show that the mechanical properties of laminates were temperature-dependent.

Temperature and moisture (hygrothermal) variation impair fiber/matrix interfacial, which plays a predominant role in matrix-dominated mechanical properties of polymer composite. Ray [16] reported on the effect of thermal and moist environments on interlaminar shear strength (ILSS) of glass/epoxy composite laminates. ILSS properties of laminates were affected by this conditioning. Jiang et al. [17] studied experimental, analytical and numerical methods on GFRP laminates to assess moisture absorption behavior used on composite bridge structures. Results confirmed that hot/wet environment significantly accelerated the moisture-induced deterioration process of materials.

Correia et al. [18] performed an experimental and analytical study on the mechanical response of GFRP pultruded profile at elevated temperatures. They used DMA and DSC tests to find glass transition temperature and decomposition process. Tensile, shear and compressive tests were occurred from 20 °C up to 250 °C to characterize the responses of GFRP material under variable temperatures. Experimental results confirmed that the mechanical performance of glass fiber-reinforced polymer material severely deteriorated at high temperatures, loaded in shear and compression, owing to the glass transition temperature of the polymer. It provided reasonably accurate estimates of experimental strength data.

Currently, the composites industry continues to evolve on renewable energy. In particular, horizontal axis wind turbine (HAWT) blades require high-performance advanced FRP composite materials. The blades were exposed to variable wind loads and environmental conditions during their lifetime [19]. With the background described above, the properties of FRP materials are temperature-dependent. The HAWT blades are composed of the skin, spar cap and shear web. Mainly, unidirectional glass was used to produce the spar cap section to withstand bending moments. The spar caps sections are exposed to fluctuating wind loads. We aimed to understand further the mechanical and damping properties of the material to use for design purposes under different environmental conditions.

This experimental study investigates the response of GFRP laminates (used for producing the spar caps section of a blade) on compressive, tensile, and viscoelastic (storage modulus, loss modulus, and damping ratio) properties as a function of temperatures and frequency. Before the test, laminates were preserved in a deep freezer at −80 °C, −20 °C, 0 °C, and room temperature (25 °C) for up to 60 days. Tensile and compressive responses of the laminates under each preserved temperature were assessed. Additionally, the storage modulus, loss modulus, damping ratio and glass transition temperature were characterized on each preserved temperature using a DMA tool. Finally, the accuracy of experimental results with empirical models was assessed to estimate the variation of storage modulus as a function of temperature and frequency. A better empirical model from the authors was proposed and its accuracy was compared with the experimental results.

## 2. Test Program

A series of unidirectional GFRP laminates were tested in compressive, tension and bending to assess their performance as a function of temperature and frequencies.

### 2.1. Materials

Unidirectional E-glass fiber, prime 27 LV epoxy resin and prime 27 LV slow hardener were purchased from AMT composites in South Africa. Table 1 shows the physical and mechanical properties of E-glass fiber and epoxy resin to prepare GFRP laminates. Matrix material was prepared with a weight mixing ratio of 10:2.6.

### 2.2. Laminate Preparation and Testing Methods

E-glass/epoxy laminates were prepared using ASTM D 3039/D 3039M, ASTM D 695 and ASTM D 5023 standards [21]. Four-ply glass fiber with a common epoxy matrix was used to prepare GFRP laminates for tensile and compressive strength testing. Twelve-ply glass fiber laminates were prepared for dynamical mechanical analysis (DMA) testing. All laminates were produced using the resin transfer molding (RTM) process. After RTM processes, laminates were cured on a glass table at 25 °C for 24 h. After these processes, laminates were post-cured in an oven at 65 °C for 16 h. Laminates were then cooled at room temperature and tabs were produced with plain weave glass fibers, using hand lay-up production techniques.

**Figure 1:**
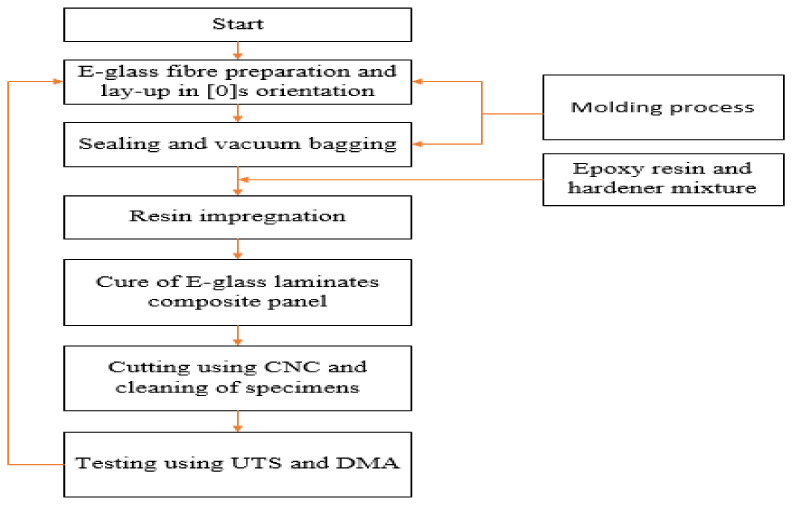
Flow chart of specimen preparation using the RTM method.

Laminates were cut using a computer numerical control (CNC) machine, with a tolerance of 0.02 mm. They were then cleaned and flashes were removed using sandpaper, before testing. Test specimens were measured, inspected for defects and placed into the composite testing laboratory for acclimatization aligned to test conditions. Matrix digestion using the burn-off method was used to determine the volume fractions according to ASTM 3171 [22]. For the present study, the volume fraction of E-glass fibers was obtained at 55%. The laminate preparing process using RTM is shown in Figure 1.

Tensile and compressive strength tests were carried out using a Lloyd LR testing machine. The testing machine was equipped with a 30 kN load cell and measurements were taken at a rate of 2 mm/min. Average tensile and compressive strength results, standard deviations, coefficients of variation and two-parameter Weibull distributions were recorded and shown in Table 2 and Table 3. Laminates were preserved in deep freezers at −80 °C, −20 °C, and 0 °C, for 60 days, to investigate and characterize the stiffness, tensile and compressive response of materials. Tensile and compressive tests were carried out at heating and testing temperatures of −80 °C, −20 °C, and 0 °C. Long-term effects of moistures were studied to investigate their effect on tensile strength, elastic modulus, failure strain and compressive strength of a material. Additional tests were carried out to determine the laminates response at 25 °C, 50 °C, 75 °C, and 100 °C. Laminates were preheated for 2 h in a binder oven, before testing to ensure that temperature was uniform along with the thickness of laminates. A heat-con thermocouple was mounted in the oven to measure temperatures during a test. An epsilon digital extensometer of 25 mm gauge length was used to measure the strain. DMA tests were carried out as per ASTM: D5023 using DMA Q 800 TA Instrument. Three-point bending modes were used. The heating rate was increased at 2 °C/min and frequencies were set at 1 Hz, 10 Hz and 100 Hz. Glass transition temperature (T_g_) of epoxy resin was measured using a DMA tool. Liquid nitrogen was used as a cooling agent. Dimensions of test samples were set at a height of 4.57 ± 0.03 mm, width 13 ± 0.02 mm and length 64 ± 0.02 mm. In DMA experiments, sensors measured the testing temperature and loading. Strain (ε) was given by [23]

(1) $$\varepsilon =\varepsilon_{0}\sin(\omega .t)$$

where $\varepsilon_{0}$ was strain amplitude, ω was the circular frequency and t denotes time. Corresponding stress σ was expressed as,

(2) $$\sigma =\sigma_{0}\sin(\omega .t+\delta )$$

where $\sigma_{0}$ was stress amplitude and δ represented phase angle between stress and strain. Storage modulus ($E^{\prime }$), loss modulus ($E^{\prime \prime }$) and damping factor (Tanδ) was expressed as,

(3) $$E^{\prime }=\left(\sigma_{0}/\varepsilon_{0}\right)\cos\delta$$

(4) $$E^{\prime \prime }=\left(\sigma_{0}/\varepsilon_{0}\right)\sin\delta$$

(5) $$\mathrm{Tan}\delta =E^{\prime \prime }/E^{\prime }$$

### 2.3. Weibull Statistical Distribution

Tensile and compressive results were analyzed by Weibull distribution, which was used to describe the strength of FRP composite materials [24]. Weibull distribution was characterized with a basic form of cumulative probability density denoted by:

(6) $$P\left(\sigma \right)=1-\exp\left[-{\left(\frac{\sigma }{\sigma_{a}}\right)}^{m}\right]\;$$

where σ  was tensile or compressive strength, $\sigma_{a}\;$ was scalar parameter (mean) and m was shape parameter. Shape parameter was obtained from tensile and compressive test data using linear fit, to linearize the form of the two-parameter Weibull probability function.

## 3. Experimental Results and Discussion

In this section, the experimental results are presented and discussed in detail with regards to compressive strength, tensile strength, and viscoelastic properties such as storage modulus, loss modulus and damping ratio as a function of temperature and frequencies. It is the known properties of epoxy resin that the force transfer capacity of the matrix between the fibers and resins is reduced as the temperature approaches the glass transition temperature. Glass fiber shows better thermal properties compared to the epoxy matrix and carried some loads on compressive and tensile directions at a higher testing temperature.

### 3.1. Compressive Tests

A compressive test was performed to assess the response of GFRP laminates as a function of temperature. Table 2 summarizes the compressive response of laminates. For the case of 0/G, compressive strength was highest and increased by 20.88%, 56.71%, 239.56% and 1744% when temperature increased. For the case of low temperature, a reduction ratio of −20/G and −80/G group of laminates were 20.44% and 21.11%. Results on compressive strength responses are greatly dependent on increasing temperature. Particularly, change in low-temperature tests occurred due to a high level of moisture swelling that initiated micro-cracks in the laminate. As shown in Table 2, the first ranged from −80 °C to 50 °C, which are temperatures below the T_g_ of the laminate. In this zone, the molecular chain mobility of the epoxy matrix did not have much change, thus, temperatures below the glass transition temperature do not significantly affect the compressive strength of GFRP laminates. The second zone contains temperatures between 50 °C and 75 °C, which is the temperatures approach to the glass transition temperature of the epoxy resin. In this zone, the epoxy resin softens, and thus, the force transfer capacity of the resin to the fiber was reduced. Due to this, the compressive strength of the laminates was decreased compared to the first zone. Above the T_g_ of the laminates at temperatures of 100 °C, the load-carrying capacity of the fiber reduced severely and obtained the least compressive strength properties.

**Figure 2:**
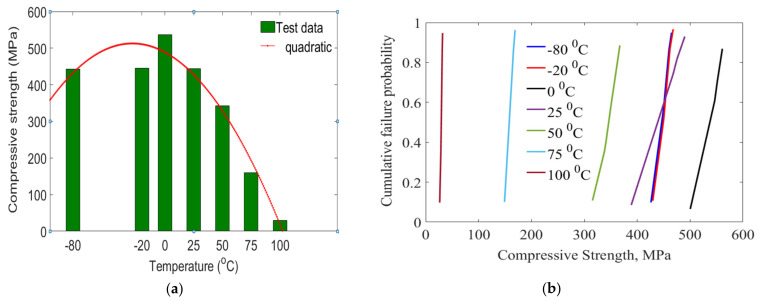
Laminates under different temperatures, (**a**) compressive strength properties (**b**) cumulative failure probability.

Compressive behavior and cumulative failure probability distribution of laminates are shown in Figure 2. Results fit well to quadratic curves. Compressive results were analyzed using the Weibull distribution model in Figure 2b. Mostly, two Weibull statistical distribution parameters, shape parameter and scaler parameter were used for characterization purposes [25]. Compressive response under different temperatures was identified from standard deviation and coefficient of variation. As presented in Table 2, model $\left(\sigma_{a}\right)$ and experimental results were within acceptable ranges of 1.28% and 3.51% variations. The correlation coefficient (R) was between 97.78% and 98.16%. Mainly, mobilization of epoxy molecules occurred as testing temperatures approached T_g_ of polymer resin. This could be the case for the reduction of the compressive response of laminates at a higher temperature. For the case of longer swelling times, laminates absorb more moisture. Consequently, van der Waals forces between polymer molecules could be lower and the hydrogen bonding may be weakened.

### 3.2. Tensile Tests

A tensile test was performed to assess the tensile response of laminates as a function of temperature. Table 3 summarizes the tensile response of laminates under variable temperatures. The highest tensile stress and stiffness response of laminates were observed at the 0 °C test. Then, tensile stresses were reduced by 27.24%, 53.28%, 239.39% and 1596.28% when the temperature increased from the 0 °C to 100 °C test. Degradation response on tensile and stiffness of laminates might happen due to plasticization and swelling effect under variable temperatures. A level of changes in strength and stiffness behavior was observed after exceeding T_g_ of the polymeric matrix. Particularly, slight degradation in strength and stiffness was observed during lower temperature tests. A high level of moisture swelling may increase the crosslinking between polymers to delay the failure of laminates. While higher degradation in mechanical properties occurred when temperatures increased. This could have happened due to a reduction of van der Waals forces and the hydrogen bond of polymer molecules. It might lead to a weakening of bonds in fiber/matrix interfaces. This would result in a reduction of compressive, tension and stiffness behaviors of laminates.

As shown in Table 3, the tensile strength and stiffness properties of GFRP laminates do not affect significantly. The reduction is severe when the temperature of 100 °C. In this case, the fiber/epoxy matrix interface was damaged significantly.

**Figure 3:**
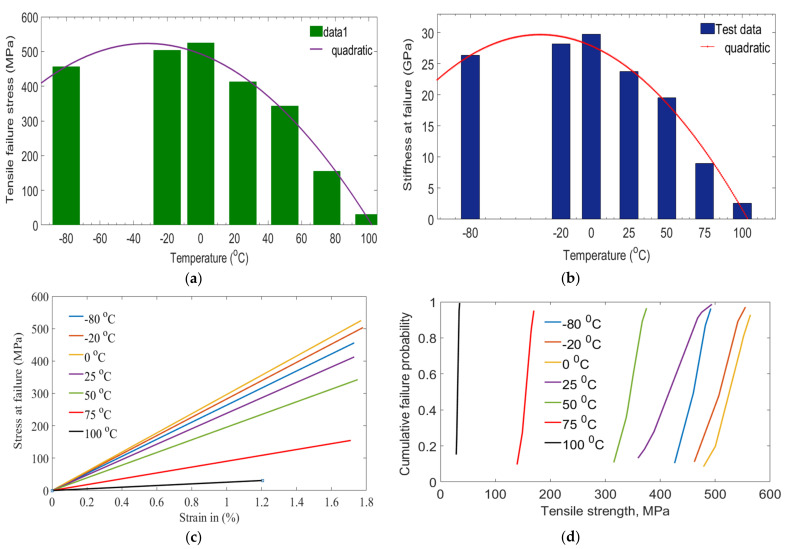
Laminates under different temperatures (**a**) tensile stress, (**b**) stiffness (**c**) stress–strain curves (**d**) cumulative failure probability.

Figure 3c plotted the stress–strain response of laminates as a function of temperature. Results indicated that the stress–strain curve was fairly lowest at the highest temperature tests. It might be occurred due to higher degradation of laminates, once the glass transition temperature of the matrix was exceeded. As shown in Table 3, the shape parameters of GFRP laminate were assessed at different temperatures. The shape parameter decreased from 25 °C up to 100 °C and increased from −20 °C up to −80 °C compared to 0 °C. This indicated the presence of scattering failure behavior between each laminate. Next, standard deviation and coefficient of variation behaviors were assessed. A 25/G laminate has the highest coefficient of variation with the lowest shape parameter. Cumulative failure probabilities of laminates under different temperatures are shown in Figure 3d.

**Figure 4:**
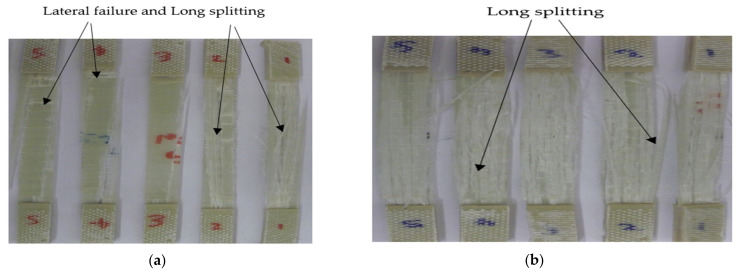
Failure modes of unidirectional GFRP laminates at testing temperatures: (**a**), −20 °C (**b**), 75 °C.

The tensile failure modes of −20/G and 75/G GFRP laminates at a testing temperature of −20 °C and 75 °C are shown in Figure 4. Lateral failure and long splitting failure modes were observed on the −20/G after tensile tests. In this case, the fiber and the epoxy matrix failed together and fractures of the fibers to the applied load direction were observed. For the case of 75/G, long splitting failure modes occurred due to softening of the epoxy resin when it approaches the glass transition temperature of the laminate.

### 3.3. DMA Tests

**Figure 5:**
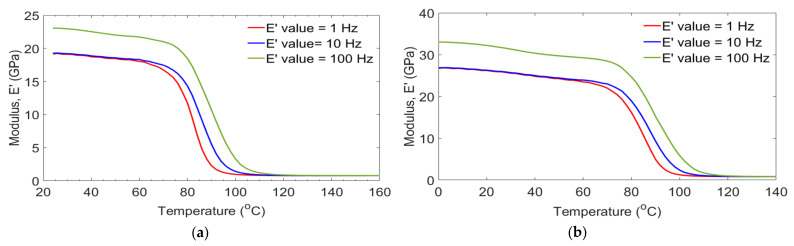

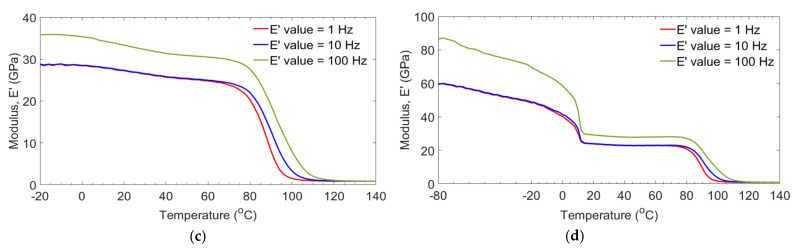
Storage modulus with temperature of GE control (**a**), 0/G (**b**), −20/G (**c**), −80/G (**d**) laminates.

Dynamic mechanical analyses were performed in GFRP laminates held at temperatures of −80 °C, −20 °C, 0 °C and room temperature (25) to assess the mechanical response as a function of temperature and frequency. Table 4 summarizes the dynamic response of GFRP laminates using the DMA tool. Figure 4 plots the storage modulus $\left(E^{\prime }\right)$ of laminates on each of the targeted temperatures and frequencies. Results show that $E^{\prime }$ behavior was reduced with increasing temperature. Mostly, $E^{\prime }$ with temperature curves provided valuable information about stiffness, degree of cross-linking and fiber/matrix interfacial bonding of the viscoelastic materials [26]. In those figures, values of $E^{\prime }$ were higher in the glass state and lower in the rubbery state. This was due to the highly immobile (frozen state) of components in the glassy region and more mobilization of polymer epoxy in the rubbery region, which did not have a closed packing arrangement. T_g_ of polymer matrix was estimated from curves of storage modulus, loss modulus and damping factor. As presented in Table 4, T_g_ values were estimated from storage modulus, loss modulus and damping factor curves. The value of $E^{\prime }$ highly decreased around 80 °C, corresponded to T_g_ of polymer resin and lower plateau to viscoelastic state. In all cases, a substantial drop in $E^{\prime }$ occurred when the temperature exceeded T_g_ values. This was occurred due to an increase in mobility of polymer chain molecules above T_g_ of resin. For the cases of Figure 5d, laminates have two glassy states before reached to a rubbery state. The first T_g_ was occurred due to prolonged moisture absorption which acts as a plasticizer that was a case on reducing hydrogen bond, Van der Waals forces and hardness of the laminates [27,28]. In those figures, the response on $E^{\prime }$ of all laminates was similar at 1 Hz and 10 Hz. While increased at 100 Hz. The similarity of response on $E^{\prime }$ of all groups of laminates at 1 Hz and 10 Hz might be due to the flow behavior of polymer matrix at low frequencies, acting similarly to flow at higher and elevated temperatures. As the frequency increased, gaps between the cross-linking of polymer matrix tended to close. This caused the material to behave in an elastic fashion. The swelling behavior of laminates was dependent on exposure to humidity. Regarding the preservation of laminates for a longer duration, may contribute to a reduction in the gaps in cross-links of the resin matrix. This could support finding the maximum $E^{\prime }$ from −80/C group laminates at 100 Hz as presented in Table 4.

**Figure 6:**
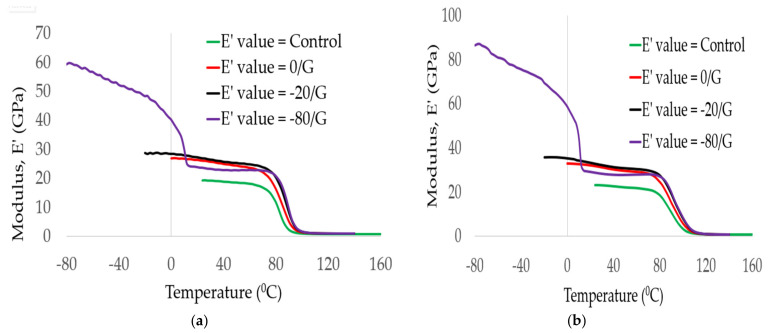
Storage modulus comparisons between GFRP laminates at the frequency (**a**), 1 Hz, (**b**), 100 Hz.

Figure 6 shows the comparison between the storage modulus results obtained at a frequency of 1 Hz and 100 Hz on control (GE), 0/G, −20/G, and −80/G laminates as a function of temperature. As can be seen from the figures, the storage modulus of all laminates increased as the frequency changed from 1 Hz to 100 Hz. Compared with storage modules of control, 0/G, −20/G, and −80/G at a testing temperature of 80 °C, the storage modulus increased by 57.45%, 52.47%, 36.15%, and 30.40% when the frequency changed from 1 to 100 Hz. The gaps between the cross-linking of the epoxy matrix might be tended to close when the frequency increases. No frequency difference between all laminates that existed below the decomposition temperature might be due to the damaging of the fiber/epoxy resin interface.

**Figure 7:**
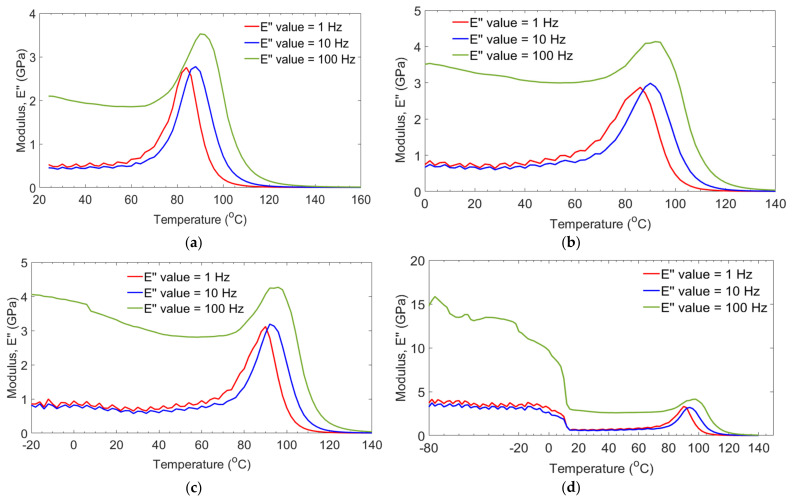
Loss modulus with temperature of GE control (**a**), 0/G (**b**), −20/G (**c**), −80/G (**d**) laminates.

Figure 7 presents loss modulus $\left(E^{\prime \prime }\right)$ properties of GFRP laminates as a function of temperature and frequency. Results presented in Figure 7a–d show that all groups of laminates have a similar response at 1 Hz and 10 Hz. Regarding the response of laminate at 100 Hz, $E^{\prime \prime }$ was highly dependent on temperature and frequency. Additionally, it was also observed that maximum $E^{\prime \prime }$ occurred at lower temperature tests for the −20/G and −80/G groups of laminates. Thus changes occurred due to the longer duration of moisture swelling which influenced the close packing arrangements of resin, resulting in increased elastically. For the case of the GE (control) group of laminate, a peak for maximum $E^{\prime \prime }$ occurred at T_g_, which was due to an increase in internal friction that enhanced mobility of polymer to dissipate heat [15]. Response in $E^{\prime \prime }$ values of −80/G laminate was highest at the first phase of glassy and GE (control) showed the lowest values. T_g_ values obtained from curves of $E^{\prime \prime }$ were higher than from $E^{\prime }$ curves. Mostly, Tg values obtained from $E^{\prime }$ curves are recommended to use for composite structural design applications.

**Figure 8:**
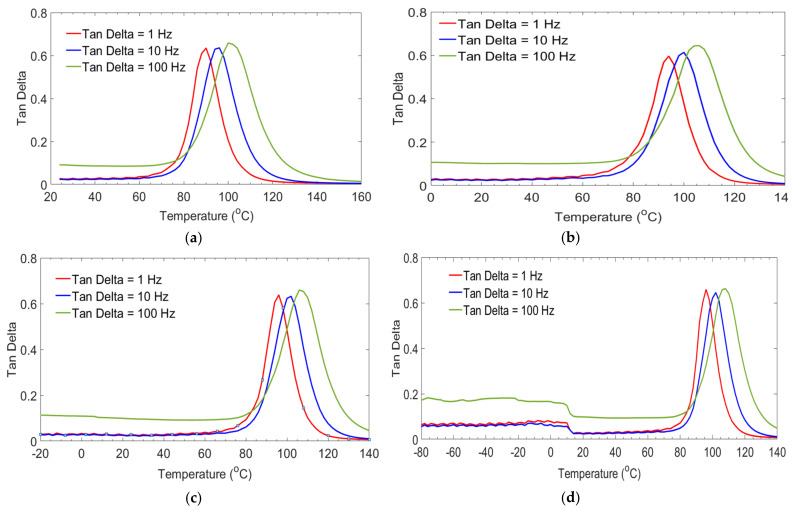
Damping factor with temperature of GE control (**a**), 0/G (**b**), −20/G (**c**), −80/G (**d**) laminates.

The damping ratio of GFRP material used for designing the spar caps parts of the blade was characterized by preserving it in different temperatures. Mainly, the spar caps section of the blade is affected by fatigue loads. We aimed to assess the damping properties of the laminates as a function of temperature and frequency. Figure 8 illustrates the response to the damping factor $\left(\tan\delta \right)$ of laminates. It was observed that values of damping behaviors of GE (control), 0/G, −20/G, and −80/G were slightly increased up to T_g_ of the epoxy resin and then reduced below the decomposition temperature of the laminates. In those figures, the damping response of all laminates was lower in the glassy region and higher in the rubbery region. The change of damping behavior at rubbery regions might be happened because of the molecular mobility of epoxy resin. Results from the damping curve illustrated different peak heights at 1 Hz, 10 Hz and 100 Hz were observed. This might occur due to closed gaps between the cross-link of epoxy resins during an increase in frequencies. T_g_ values of all groups of laminates were assessed on each targeted temperature and frequency. Results indicated that T_g_ of the resin shifted to higher temperatures as the frequencies changed from 1 Hz to 100 Hz. An increase in T_g_ occurred because of temperature-dependent molecular relaxation behavior in polymer material. Peak height and damping behavior had a direct relationship with fiber/matrix interface strength.

**Figure 9:**
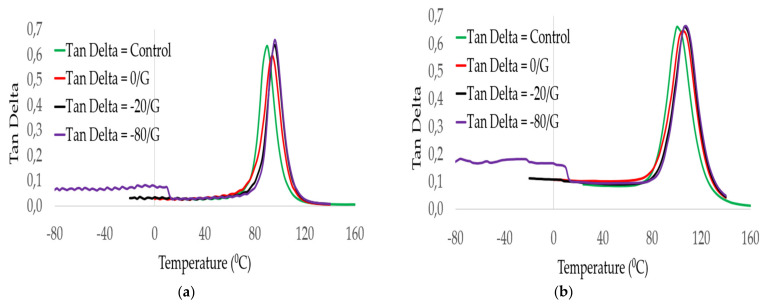
Damping ratio comparisons between GFRP laminates at a frequency (**a**), 1 Hz, (**b**), 100 Hz.

The damping ratio of all GFRP laminates was compared at frequencies of 1 and 100 Hz as shown in Figure 9. Results indicated that GFRP laminates preserved at 0 °C and −80 °C for 60 days each of the weakest and highest damping ratios were obtained.

## 4. Statistical Analyses

### Analysis of Variance (ANOVA)

To assess the effect of temperature on compressive and tensile strength of GFRP composite laminates using one-way analysis of variance (ANOVA) was performed [29]. The ANOVA results of the compressive strength and the tensile strength test performed on laminates under lower and higher testing temperatures are presented in Table 5 and Table 6. The SS is the sum of the square of the deviations of all observations from their mean; df is the number of degrees of freedom; MS is the mean square which was obtained by dividing SS by the respective degree of freedom; F is the variation between sample mean (Mean Square Between) to the variation within the samples (Mean square Error); P is probability value; F crit is an indicator corresponds to the *p* values for which, when F crit < F indicated that the variables made a significant effect on the outcomes. As shown in Table 5 and Table 6, *p* < 0.05 (F crit <F) was obtained for compressive and tensile strength of GFRP laminate tested under temperatures of −80–100 °C. It indicated that the contribution of temperature is statistically significant and must be considered when evaluating the compressive and tensile strength properties of GFRP material under variable temperatures.

## 5. Comparison between the Prediction Models and Experimental Results

In the present section, experimental results obtained from DMA tests were used to access the accuracy of models suggested by authors on the storage modulus behavior of GFRP laminates as a function of temperature and frequency. A review of the literature showed that there was a gap in research work on empirical models. Yu Bai et al. [23] summarized the currently available work on the subject which gave information on the degradation of mechanical properties of FRP materials subject to various thermal loadings. According to Gibson et al. [30], variation of mechanical behavior of FRP material as a function of temperature can be determined based on the following equations.

(7) $$P\left(T\right)=\frac{P_{U}+P_{R}}{2}-\frac{P_{U}-P_{R}}{2}\tanh(k\left(T-T^{\prime }\right))\;$$

where P(T) was elastic modulus at a specified temperature T, P_U_ was elastic modulus at room temperature (before transition), P_R_ was material relaxed modulus before decomposition (after transition). $k\;\mathrm{and}\;T^{\prime }$ were variables identified by fitting data using regression analysis. Value of $T^{\prime }$ was recommended when elastic modulus fell rapidly (assumed Tg values).

An empirical model on the temperature-dependent elastic modulus of FRP materials was proposed by Gu and Asaro [31], considering the following degradation relation, which was determined by fitting a curve to the experimental data. It was given by:

(8) $$P\left(T\right)=P_{U}{\left(1-\frac{T-T_{r}}{T_{\mathrm{ref}}-T_{r}}\right)}^{g}\;$$

where $T_{\mathrm{ref}}\;$ was the temperature at which elastic modulus tends to zero value. $T_{r}$ was ambient temperature and g  was a power-law index between 0 and 1.

Mahieux and Reifsnider [32] predicted the instantaneous degradation of stiffness of FRP composite, with temperatures (in Kelvin) which have effects of breaking, relaxing and increasing intermolecular bonds in a polymeric matrix. Their expression was given

(9) $$P\left(T\right)=P_{R}+\left(P_{U}-P_{R}\right)\exp\left(-{\left(\frac{T}{T_{0}}\right)}^{n}\right)\;$$

$T_{0}\;$ was relaxation temperature and n  was Weibull exponent. Acceptable fitting of property data can be assessed with several possible n values in the range of 15–21. The coefficients k,g and n were determined using an excel solver. A regression analysis was carried out to achieve a minimum error value between experimental results and empirical models given in Equations (7)–(9). Based on regression analysis, a best fit empirical model can be assessed as a function of temperature and frequency.

**Figure 10:**
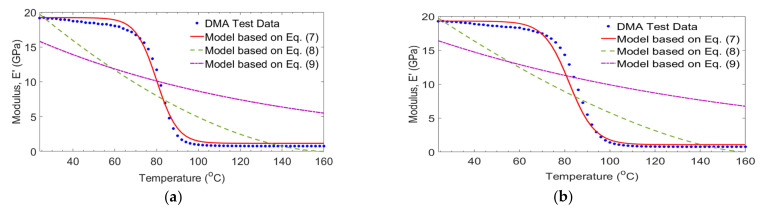

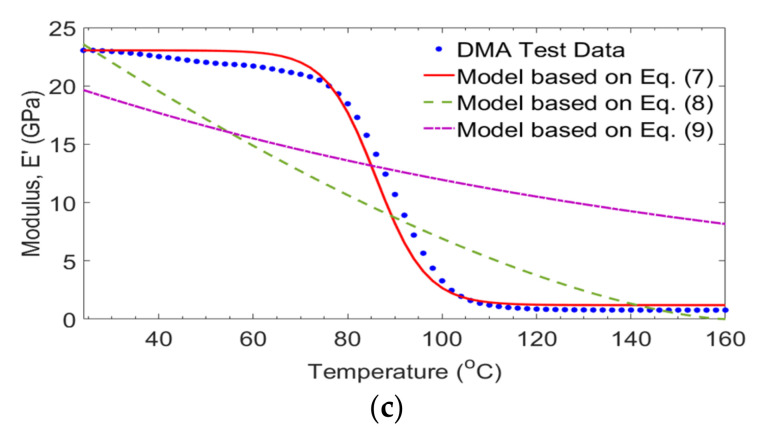
Comparison of test and empirical models for GE (control) laminates at frequencies of 1 Hz (**a**), 10 Hz (**b**) and 100 Hz (**c**).

Figure 10 shows the comparison between the storage modulus results of GE (control) laminates and those predicated by other researchers [30,31,32]. Based on the work of Gu and Asaro [31] and Mahieux and Reifsnider [32] the prediction equations are not appropriate to predicate the storage modulus. The minimum square errors were between 13.51% and 15.08%. While the Gibson et al., [30] equation is more accurate. The errors were between 0.35% and 0.78% as the frequencies changed from 1 Hz to 100 Hz.

**Figure 11:**
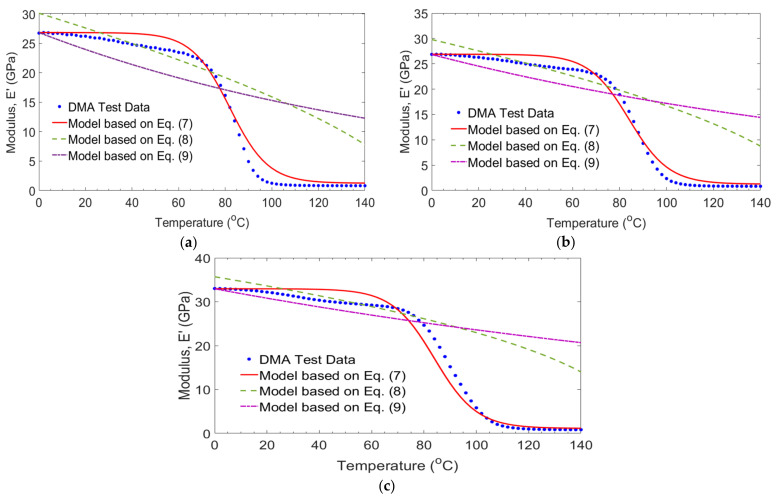
Comparison of measured and empirical models for 0/G laminates at frequencies of 1 Hz (**a**), 10 Hz (**b**) and 100 Hz (**c**).

Figure 11 plots the comparison between the storage modules results of 0/G laminates with the predicated model developed by Gibson et al. [30], Gu and Asaro [31], and Mahieux and Reifsnider [32] subjected to temperature and frequency. As can be seen from the Figure, the equations developed by Gu and Asaro [31] and Mahieux and Reifsnider [32] are not appropriate to the prediction of the storage modulus for the temperature exceeding 80 °C. The minimum square errors were between 12.90% and 18.06%. While the Gibson et al. [30] equation is more accurate for prediction in this temperature range. The Errors were between 0.81% and 1.91% as the frequencies changed from 1 Hz to 100 Hz.

**Figure 12:**
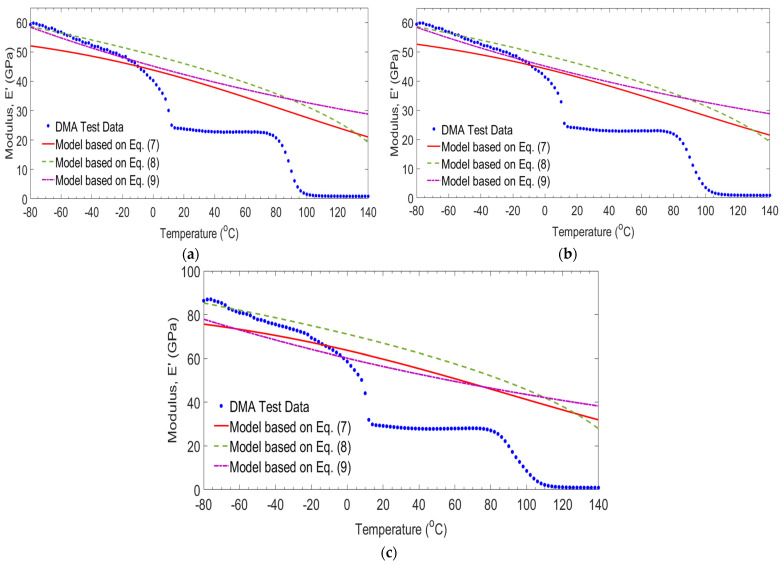
Comparison of measured and empirical models for −20/G laminates at frequencies of 1 Hz (**a**), 10 Hz (**b**) and 100 Hz (**c**).

The comparison between the storage modules results of −20/G laminates obtained in the study and those empirical models developed by Gibson et al. [30], Gu and Asaro [31], and Mahieux and Reifsnider [32] as a function of temperature and frequency are shown in Figure 12. The equations Gu and Asaro [31] and Mahieux and Reifsnider [32] seems accurate for temperature below 80 °C. While this equation does not predict the storage modulus when the temperature exceeds 88 °C. The errors using those empirical models were between 14.17% and 22.59%. The empirical model given by Gibson et al. [30] is a close correlation with test results. Errors with the experimental results were between 1.78% and 2.56% as the frequencies changed from 1 Hz to 100 Hz.

**Figure 13:**
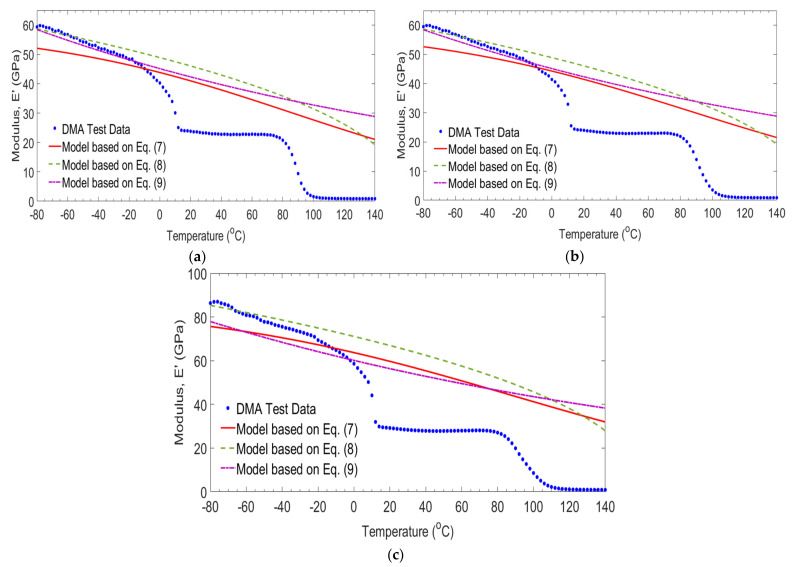
Comparison of measured and empirical models for −80/G laminates at frequencies of 1 Hz (**a**), 10 Hz (**b**) and 100 Hz (**c**).

Figure 13 illustrates the comparison between the storage modulus result of −80/G laminates obtained from this study and those empirical models developed by Gibson et al. [30], Gu and Asaro [31], and Mahieux and Reifsnider [32] as a function of temperature and frequency. In this case, the equations developed by Gu and Asaro [31], and Mahieux and Reifsnider [32] are not appropriate to predict the storage modulus for temperatures exceeding −14 °C. The minimum square errors using Gu and Asaro [31] and Mahieux and Reifsnider [32] are between 5.62% and 9.80%. Whereas, based on Gibson et al. [30], the minimum square error was between 4.70% and 7.36% as the frequencies changed from 1 Hz to 100 Hz.

Generally, the equation developed by Gibson et al. [30] is accurate to predict the storage modulus of the GFRP laminates considered in this study. However, further studies are necessary to revise to obtain more accurate relations with other empirical models.

### Proposed Empirical Models for Experimental Results

Based on least square regression analyses, the empirical model developed by Gibson et al. [30] was proposed for predicting storage modulus results of GE (control), 0/G, −20/G and −80/G laminates on each targeted temperature and frequency. Values of coefficients were determined by calibrating the test data and predicted model intending to achieve a minimum square error. These parameters for each laminate are given in Table 7.

## 6. Conclusions

This study is part of ongoing research to assess the lifetime and performance of FRP material available on the structural design of wind turbine blades applicable on colder and hotter area wind farms. This paper studied the compressive strength, tensile strength, tensile modulus, and viscoelastic properties (storage modulus, loss modulus, and damping ratio) of different types of GFRP laminates after being exposed to lower and higher temperatures. Based on experimental results, the following observations and conclusions were drawn:

1. Temperatures ranging from −80 °C to 50 °C, which were below the glass transition temperature of unidirectional GFRP laminates, did not significantly affect the compressive strength, tensile strength, and stiffness properties. This indicated that temperatures below T_g_ of GFRP laminates slightly affect their mechanical performances. In this case, long splitting and lateral failure mode have occurred. The load transfer capacity of the epoxy resin to the fiber was strong.
2. At a testing temperature of 75 °C, which was near to T_g_ of GFRP laminates, the compressive strength, tensile strength, and stiffness properties were decreased due to softening of the epoxy matrix, which was weak to transfer the load to the fibers. In this case, long splitting types of failure mode have occurred.
3. At a testing temperature of 100 °C, which was above the T_g_ of GFRP laminates, the compressive strength, tensile strength, and stiffness properties were decreased significantly due to the damaging of the fiber/matrix interface. In this case, the load-carrying capacity of the fiber was reduced severely.
4. The storage modulus of all groups of laminates decreased as the temperature increased. It happened due to the higher mobilization on the rubbery region of the epoxy resin, which lost their closed packing arrangements. The highest storage modulus values on −80/G laminate were observed on the first phase of the glassy region at 100 Hz. This first T_g_ occurred due to the prolonged absorption of moisture, which acts as a plasticizer for the laminates to reduce their bonds and hardness.

The experimental results presented in this study provide a better understanding of the viscoelastic, compressive, and tensile strength degradation of GFRP laminates under lower and higher temperatures. However, more work is needed to validate the experimental results with the predicted models. Additionally, it was observed that 0/G laminates had the lowest and −80/G had the largest damping ratio as a function of temperature and frequency. Finally, these experimental results can be input to understand the behavior of different types of GFRP laminates used for the production of spar cap components of the blade.

## Figures and Tables

**Table 1:** Properties of E-glass fiber and epoxy resin [20].

Materials	Stiffness [GPa]	Tensile Strength [MPa]	Density [kg/m^3^]	Poisson’s Ratio
E-glass	72.5	2350	2570	0.25
Epoxy resin	3.3	69.9	1020	0.36

**Table 2:** Compressive properties of laminates at various temperatures.

Designation of GFRP Laminates	$\sigma_{a}(\mathrm{MPa})$	m Values	R Values	Compressive Strength (MPa)	Standard Deviation and Coefficient of Variation
−80/G	451.93	38.37	97.91%	443.19	(24.15, 5.45%)
−20/G	453.74	39.50	97.78%	445.68	(23.07, 5.18%)
0/G	547.78	29.69	97.85%	536.77	(24.36, 4.54%)
25/G	458.36	14.65	98.16%	444.04	(48.08, 10.83%)
50/G	352.83	19.36	98.04%	342.52	(18.92, 5.52%)
75/G	161.82	27.40	97.78%	159.77	(9.24, 5.78%)
100/G	30.12	17.85	98.14%	29.10	(2.02, 6.94%)

**Table 3:** Tensile strength and modulus of laminates at various testing temperatures.

Designation of GFRP Laminates	$\sigma_{a}(\mathrm{MPa})$	m Values	R Values	Tensile Strength (MPa)	Standard Deviation and Coefficient of Variation (Tensile)	Tensile Modulus (GPa)	Standard Deviation and Coefficient of Variation (Modulus)
−80/G	467.79	23.65	97.94%	456.16	(28.22, 6.19%)	26.36	(1.09, 4.14%)
−20/G	518.40	18.56	98.04%	503.25	(39.71, 7.89%)	28.17	(1.95, 6.92%)
0/G	538.24	20.69	98.01%	525.16	(33.45, 6.37%)	29.73	(1.78, 5.99%)
25/G	431.16	10.75	98.35%	412.60	(55.01, 3.33%)	23.73	(2.33, 9.81%)
50/G	352.51	19.53	98.07%	342.52	(18.92, 5.52%)	19.51	(1.06, 5.43%)
75/G	159.35	17.16	98.09%	154.69	(12.64, 8.17%)	9.00	(0.53, 5.89%)
100/G	31.89	17.61	98.01%	30.95	(2.28, 7.37%)	2.58	(0.29, 1.24%)

**Table 4:** Maximum storage modulus, loss modulus, the peak of tanδ and T_g_ of laminates.

Glass/Epoxy Laminates	${E^{\prime }}_{\max}(\mathrm{GPa})$	$E^{\prime }\mathrm{at}Tg_{\;}(\mathrm{GPa})$	$E^{\prime \prime }\;\mathrm{at}\;\mathrm{Start}\;(\mathrm{GPa})$	$E^{\prime \prime }\;\mathrm{at}T_{g}$ (GPa)	Peak Height on tanδCurve	$T_{g}\;\mathrm{on}\;\tan\delta$(°C)	$T_{g}\;\mathrm{on}\;E^{\prime }({}^{\circ}C)$	$T_{g}\;\mathrm{on}\;E^{\prime \prime }$ (°C)
GE (1 Hz)	19.22	11.73	0.54	2.76	0.635	90	80	84
GE (10 Hz)	19.29	12.89	0.45	2.78	0.636	96	82	88
GE (100 Hz)	23.05	15.76	2.09	3.53	0.658	100	84	90
0/G (1 Hz)	26.84	14.12	0.85	2.88	0.596	94	82	86
0/G (10 Hz)	26.88	15.60	0.74	2.99	0.613	98	84	90
0/G (100 Hz)	33.02	21.54	3.52	4.14	0.645	106	84	92
−20/G (1 Hz)	28.74	18.57	0.92	2.97	0.639	96	82	88
−20/G (10 Hz)	28.82	17.41	0.82	3.19	0.632	102	86	92
−20/G (100 Hz)	35.89	23.20	4.06	4.27	0.660	106	86	96
−80/G (1 Hz)	59.78	15.87	4.13	3.31	0.659	96	86	90
−80/G (10 Hz)	59.88	18.60	3.72	3.20	0.645	102	86	94
−80/G (100 Hz)	87.00	22.20	15.85	4.15	0.662	108	88	98

**Table 5:** Resulting ANOVA for compressive strength of GFRP laminates tested under temperatures of −80–100 °C.

Source of Variation	SS	df	MS	F	*p*-Value	F Crit
Between Groups	1,002,600.17	6	167,100.03	512.11	$2.83\times 10^{-27}$	2.45
Within Groups	9136.4	28	326.3			
Total	1,011,736.57	34				

**Table 6:** Resulting ANOVA for tensile strength of GFRP laminates tested under temperatures of −80−100 °C.

Source of Variation	SS	df	MS	F	*p*-Value	F Crit
Between Groups	1,047,489.77	6	174,581.63	209.08	$6.44\times 10^{-22}$	2.44
Within Groups	23,379.2	28	834.97			
Total	1,070,868.97	34				

**Table 7:** Coefficients based on Gibson et al. [30] curve-fitting on experimental results [33].

Frequency	Coefficient	GE (control)	0/G	−20/G	−80/G
1 Hz	P_u_ (GPa)	19.22	26.84	28.79	59.00
	P_r_ (GPa)	1.16	1.25	1.15	1.17
	k	0.1	0.061	0.062	0.004
	T’	80	82	82	86
	R	0.9982	0.9959	0.9911	0.9761
10 Hz	P_u_ (GPa)	19.29	26.88	28.87	59.88
	P_r_ (GPa)	1.08	1.25	1.17	1.21
	k	0.089	0.059	0.072	0.0059
	T’	82	84	86	86
	R	0.9953	0.9956	0.9934	0.9718
100 Hz	P_u_ (GPa)	23.05	32.96	35.89	87.00
	P_r_ (GPa)	1.20	1.14	1.15	1.19
	k	0.094	0.062	0.060	0.0056
	T′	86	84	86	88
	R	0.9960	0.9904	0.9871	0.9625

## Data Availability

Data presented in this study are available in the article.
